# Circulating Cancer Stem Cell-Derived Extracellular Vesicles as a Novel Biomarker for Clinical Outcome Evaluation

**DOI:** 10.1155/2019/5879616

**Published:** 2019-11-18

**Authors:** D. Brocco, P. Lanuti, P. Simeone, G. Bologna, D. Pieragostino, M. C. Cufaro, V. Graziano, M. Peri, P. Di Marino, M. De Tursi, A. Grassadonia, I. G. Rapposelli, L. Pierdomenico, E. Ercolino, F. Ciccocioppo, P. Del Boccio, M. Marchisio, C. Natoli, S. Miscia, N. Tinari

**Affiliations:** ^1^Clinical Oncology Unit, SS Annunziata Hospital, Chieti, Italy; ^2^Department of Medicine and Aging Sciences, University “G. d'Annunzio” of Chieti-Pescara, Chieti, Italy; ^3^Centre on Aging Sciences and Translational Medicine (Ce.S.I.-Me.T.), University “G. D'Annunzio” of Chieti-Pescara, Chieti, Italy; ^4^Department of Medical, Oral and Biotechnological Sciences, University “G. D'Annunzio” of Chieti-Pescara, Analytical Biochemistry and Proteomics Laboratory, Chieti, Italy; ^5^Cancer Research UK Cambridge Institute, University of Cambridge, Cambridge CB2 0RE, UK; ^6^Department of Medical, Oral and Biotechnological Sciences, Gabriele D'Annunzio University, Chieti, Italy; ^7^Department of Medical Oncology, Istituto Scientifico Romagnolo per lo Studio e la Cura dei Tumori (IRST) IRCCS, Meldola, Italy

## Abstract

The recent introduction of the “precision medicine” concept in oncology pushed cancer research to focus on dynamic measurable biomarkers able to predict responses to novel anticancer therapies in order to improve clinical outcomes. Recently, the involvement of extracellular vesicles (EVs) in cancer pathophysiology has been described, and given their release from all cell types under specific stimuli, EVs have also been proposed as potential biomarkers in cancer. Among the techniques used to study EVs, flow cytometry has a high clinical potential. Here, we have applied a recently developed and simplified flow cytometry method for circulating EV enumeration, subtyping, and isolation from a large cohort of metastatic and locally advanced nonhaematological cancer patients (*N* = 106); samples from gender- and age-matched healthy volunteers were also analysed. A large spectrum of cancer-related markers was used to analyse differences in terms of peripheral blood circulating EV phenotypes between patients and healthy volunteers, as well as their correlation to clinical outcomes. Finally, EVs from patients and controls were isolated by fluorescence-activated cell sorting, and their protein cargoes were analysed by proteomics. Results demonstrated that EV counts were significantly higher in cancer patients than in healthy volunteers, as previously reported. More interestingly, results also demonstrated that cancer patients presented higher concentrations of circulating CD31+ endothelial-derived and tumour cancer stem cell-derived CD133 + CD326- EVs, when compared to healthy volunteers. Furthermore, higher levels of CD133 + CD326− EVs showed a significant correlation with a poor overall survival. Additionally, proteomics analysis of EV cargoes demonstrated disparities in terms of protein content and function between circulating EVs in cancer patients and healthy controls. Overall, our data strongly suggest that blood circulating cancer stem cell-derived EVs may have a role as a diagnostic and prognostic biomarker in cancer.

## 1. Introduction

The oncological therapies have profoundly changed in the last years, due to a better comprehension of the biological processes leading to tumour development and progression. Old therapeutic paradigms have been overcome by the concept of “precision medicine,” which aims for the administration of tailored therapies. Accordingly, cancer patients greatly benefit from the availability of novel tissue and blood biomarkers able to better predict responses to novel anticancer therapeutics and improve clinical outcomes in selected patient populations. Nevertheless, the diagnosis, treatment, and follow-up of cancer patients still suffer from the lack of dynamic measurable indicators of tumour pathologic processes and pharmacological responses.

The last decades have seen a growing interest in the involvement of extracellular vesicles (EVs) in cancer physiopathology, and their potential role as cancer biomarkers has been underlined [1–3].

EVs are particles naturally delivered into the extracellular microenvironment, containing a rich cargo of DNA, RNA, miRNAs, proteins, lipids, and metabolites [4, 5]. Three main subtypes of EVs have been described, based on their size and biogenesis: exosomes, ectosomes, also known as microvesicles (MVs) or microparticles, and apoptotic bodies. Exosomes originate from the endosomal system, and their diameter ranges from 30 to 150 nm. EVs are released by outward budding of the plasma membrane and measure 100 to 1000 nm in diameter. Apoptotic bodies, which are produced by cells destined to programmed cell death, are heterogeneous in size with a diameter ranging from 200 to 5000 nm [6].

Several studies have described the role of EVs as mediators in the intercellular crosstalk for both short- and longer-distance signalling [7–12]. Moreover, the transfer of molecular cargoes promotes different target cell responses, modifying the microenvironment and modulating the immunological machinery [13].

It has been demonstrated that EVs are involved in the pathogenesis of a number of diseases, including cancer [2, 9, 14, 15]. Both *in vitro* and *in vivo* studies have elucidated the active role of EVs in cancer biology. In particular, EVs participate in angiogenesis, tumour progression and metastasis, tumour-stroma interactions, and further biological processes [16–23]. Several evidences suggest that tumour cells produce higher numbers of EVs as compared with nonmalignant cells [24].

Interestingly, tumour-derived EVs harbour an enriched protein and genetic cargo when compared with EVs derived from normal cells [25, 26]. Based on these observations, peripheral blood circulating EVs can be recognised as a flourishing source of potential biomarkers [27–31] and, in this context, a phenotypical characterisation of blood circulating tumour-derived EVs, based on the analysis of cancer-related surface protein expression has been attempted [32–34]. Furthermore, recent *in vivo* studies have demonstrated a possible prognostic and predictive role of EV subtypes in cancer patients [34–37].

Currently, researchers are producing a huge effort for the identification of new disease-related EV phenotypes, possibly useful for the development of new therapeutic approaches [38, 39]. Indeed, larger EVs can be easily isolated from peripheral blood and characterized by multiple techniques, such as flow cytometry [7–10, 14]. For this reason, the identification and characterisation of peripheral blood circulating cancer-related EVs have been proposed as a new method of liquid biopsy, which possibly allows to avoid the more invasive tissue biopsy, to extend the benefits of molecular characterization to early diagnosis, and to monitor temporal and spatial heterogeneity of tumour cells.

Given the increasing relevance of this research field, we carried out an observational prospective study, in order to shed light on the role of tumour-derived EVs, both as diagnostic and prognostic markers in cancer patients. We focused on flow cytometry identification and proteomics characterisation of peripheral blood circulating EVs with the aim to identify new possible markers to detect and characterise circulating cancer-related EV subpopulations through a comparative analysis of EV subtypes in metastatic cancer patients and healthy volunteers. Finally, these findings have been correlated with the clinical outcomes of patients, in order to explore the potential prognostic and predictive role of EVs.

## 2. Materials and Methods

### 2.1. Patients

This observational prospective study was approved by the local ethics committee. All subjects involved in the study gave a written informed consent. Peripheral blood (PB) samples were obtained from 106 metastatic and locally advanced nonhaematological cancer patients and 25 healthy volunteers, recruited from the Clinical Oncology Unit (“SS. Annunziata” Hospital, Chieti, Italy). The demographic characteristics of all enrolled subjects were summarized in Supplementary [Supplementary-material supplementary-material-1]. Samples were collected at the baseline, before the first or the subsequent cancer treatment lines, and at the time of the first radiological assessment. PB samples were collected both for patients and for healthy volunteers in the same conditions.

### 2.2. Extracellular Vesicle Staining for Flow Cytometry

From each enrolled subject, two sodium citrate tubes (Becton Dickinson Biosciences (BD), San Jose, CA, USA, Ref 454387) were used to collect the peripheral blood samples, using 21 G needles. Samples were processed within 4 hours from bleeding. The first harvested tube of PB was discarded to minimize venepuncture-induced vascular damage effects [40, 41]. Of note, phalloidin was added to the reagent mix to stain events characterized by damaged membranes, given its binding to F-actin [10, 42]. The staining was performed following an already described protocol [10]. In detail, as the first step, the reagent mix was prepared by adding FITC-conjugated phalloidin (when needed) and LCD (BD Biosciences–Catalogue, #626267, Custom Kit), and all reagents detailed in Table 1 (Panel 1 or Panel 2 or Panel 3) were added to 195 *μ*l of PBS 1X, and then 5 *μ*l of whole blood was added to the mix. After 45 min of staining (RT, in the dark), 500 *μ*l PBS 1X was added to each tube, and 1 × 10 [6] events/sample were recorded by flow cytometry (FACSVerse, BD Biosciences).

To avoid the immune complex formation and the unspecific background linked to the antibody aggregation, each antibody stock solution was centrifuged before its use, at 21,000 *g* for 12 minutes.

### 2.3. Extracellular Vesicle Flow Cytometry Acquisition

The trigger threshold was set on the channel in which the LCD emits (allophycocyanin (APC) channel; threshold value = 200/262,144), while in order to avoid the loss of the events of interest, no threshold on scatter parameters was applied. The signal pulse height (H) was measured and represented for the forward scatter (FSC), the side scatter (SSC), and any fluorescent signal. EV scatter properties were established and validated by the Rosetta Calibration System (Exometry, Amsterdam, NL), as previously described [43], and by running Megamix-Plus beads (Byocitex, Marseille, France) at the same photomultiplier (PMT) amplification used for EV detection. Each antibody/reagent used in the panels was titrated (8-point titration) under the assay conditions; dilutions were established based on achieving the optimal signal to noise ratio [44]. The evaluation of nonspecific fluorescence was obtained by acquiring Fluorescence Minus One (FMO) and isotype controls [45, 46].

Reagent-only and buffer-only controls were also analysed, and we observed that establishing the APC channel trigger threshold as mentioned above, in both cases, produced the acquisition of almost no events during the time interval needed for the sample acquisition (∼1 minute). A sample treated by a solution of 1% Triton X-100 was acquired in order to verify that LCD staining targets intact EVs.

Compensation was assessed using CompBeads (BD) and single-stained fluorescent samples. Data were analysed using FACSDiva v 6.1.3 (BD), FACSuite v 1.0.6.5230 (BD), and FlowJo v 10 (TreeStar, Ashland, OR, USA) software. Extracellular vesicle numbers were obtained by the volumetric count. This polychromatic flow cytometry method allowing the analysis of EV concentrations and subtyping has been recently patent submitted (European patent application number EP19164567.0).

### 2.4. Gating Strategy for Extracellular Analysis and Subtyping

#### 2.4.1. Panel 1

Supplementary [Supplementary-material supplementary-material-1] shows a dotplot representing the SSC-H and the FSC-H, used to set a region under the one in which platelets (PLTs) fall. Such a region was defined as a “platelet-free area.” Events of the “platelet-free area” were then represented on an LCD-H/Phalloidin-H dotplot, and EVs were identified as LCD-positive/phalloidin-negative dots (Supplementary [Supplementary-material supplementary-material-1]). Therefore, EVs (LCD+/phalloidin−events) were analysed on a CD45-H/CD133-H dotplot, and CD45 + events were gated (Supplementary [Supplementary-material supplementary-material-1]). A CD45-negative logical gate was set, and the resulting population was plotted on a CD326-H/CD133-H dotplot (Supplementary [Supplementary-material supplementary-material-1]). Several EV phenotypes were here identified (CD133+/CD326-; CD133+/CD326+; CD133-/CD326+).

#### 2.4.2. Panel 2

EVs were identified as LCD-positive/phalloidin-negative events, falling in the “platelet-free area,” as described in Supplemental [Supplementary-material supplementary-material-1]-[Supplementary-material supplementary-material-1]. EVs were then represented on a CD31-H/CD41a-H dotplot (Supplementary [Supplementary-material supplementary-material-1]), and events showing the CD31+/CD41a + phenotype were identified as platelet-derived EVs (PLT-EVs). A PLT-EVs-negative logical gate was set, and the resulting population was plotted on a CD45-H/CD31-H dotplot (Supplementary [Supplementary-material supplementary-material-1]). CD45 + events were identified as leukocyte-derived EVs, while the CD31+/CD45− compartment was defined as the endothelial-derived EV population.

#### 2.4.3. Panel 3

Events of the “platelet-free area” were identified as described in Supplementary [Supplementary-material supplementary-material-1] and then represented on an LCD-H/CD235a-H dotplot; given that the majority of phalloidin + events falling in this area results in CD235a+ (not shown), here EVs were identified as LCD-positive/CD235a-negative dots (Supplementary [Supplementary-material supplementary-material-1]). Those events were analysed on a CD45-H/CD90-H dotplot, and CD45+ events were gated (Supplementary [Supplementary-material supplementary-material-1]). A CD45-negative logical gate was set, and the resulting population was plotted on a CD29-H/CD90-H dotplot (Supplementary [Supplementary-material supplementary-material-1]). Several EV phenotypes were here identified (CD90+/CD29-; CD90+/CD29+; CD90-/CD29+).

### 2.5. Extracellular Vesicle Separation by Fluorescence-Activated Cell Sorting

Extracellular vesicles were separated (100 *μ*m nozzle) by using a FACSAria III cell sorter (BD Biosciences) from whole peripheral blood samples on the basis of their positivity to the LCD and negativity to phalloidin, combined with their SSC-H and FSC-H features. The postsorting purity was assessed by reanalysing purified samples, as recommended, and purity was constantly higher than 90% [47]. As recently published, the EV separation method described here allowed the obtainment of EV preparations that resulted free from soluble circulating contaminants that usually affect EV samples purified using state-of-the-art techniques (i.e., ultracentrifugation) [10].

### 2.6. Extracellular Vesicle Label-Free Proteomics

Two million pooled purified EVs from lung cancer patients were employed for proteomics investigation. As already published, the number of separated EVs (obtained by the counting performed using the fluorescent-activated cell sorter) can be used as a normalization parameter for proteomics analyses [7, 10]. A typical digestion protocol of filter-aided sample preparation (FASP) was carried out overnight at 37°C using trypsin (Promega, Madison, WI). EV-digested proteins from each sample were analysed in triplicate by liquid chromatography tandem mass spectrometry (LC-MS/MS), using a Proxeon EASY-nLCII (Thermo Fisher Scientific, Milan, Italy) chromatographic system coupled to a Maxis HD UHR-TOF (BrukerDaltonics GmbH, Bremen, Germany) mass spectrometer. Peptides were loaded on the trapping EASY-Column C18 (2 cm L, 100 *μ*m ID, 5 *μ*m ps, Thermo Fisher Scientific) and then separated on an Acclaim PepMap100 C18 (75 *μ*m ID, 25 cm L, 5 *μ*m ps, Thermo Fisher Scientific) nanoscale chromatographic column. The flow rate was set at 300 nL/min, with a total run time of 90 minutes, as already described [7]. The mass spectrometer was operated in positive-ion polarity and auto MS/MS mode (Data Dependent Acquisition(DDA)), using N_2_ as collision gas for CID fragmentation. Precursors in the range 350 to 2,200 *m*/*z* (excluding 1,220.0–1,224.5 *m*/*z*) with a preferred charge state from +2 to +5 (excluding singly charged ions) and absolute intensity above 4,706 counts were selected for fragmentation in a maximum cycle time of 3 seconds. Precursors were actively excluded from selection for 30 seconds after acquiring. Isolation width and collision energy for MS/MS fragmentation were set according to the mass and charge state of the precursor ions, with in-source reference lock mass (1,221.9906 *m*/*z*) online acquisition, throughout the runs.

### 2.7. Data Processing of Label-Free Proteomics Analysis

Quantitative data analysis was performed by a free computational proteomics platform, MaxQuant version 1.3.3.4. (Max-Planck Institute for Biochemistry, Martinsried, Germany), using the raw data file of MS/MS spectra. Peak lists, generated in MaxQuant, were searched using Andromeda [48] peptide search engine against the UniProt database (released 2018_04, taxonomy *Homo sapiens*; 20,874 protein entries) supplemented with frequently observed contaminants and containing forward and reverse sequences. Multiplicity was set to one because a label-free quantification was performed. Trypsin digestion mode was specified with up to two missed cleavages. Carbamidomethylation of cysteines (C) was defined as fixed modification and used in protein quantification, while oxidation of methionines (M) was set as variable modification. Minimum peptide length of 7 amino acids was set, and the search space was limited to a maximum peptide mass of 4600 Da. MaxQuant uses individual mass tolerances for each peptide; the initial maximum precursor mass tolerances were set by default to 0.07 Da in the first search and 0.006 Da in the main search, and the fragment mass tolerance was set to 0.1 Da. A retention time tolerance of 2 min was used to align any time shift in acquisition between samples. False discovery rate (FDR) at the protein level was set at 2%, while at the peptide level was set at 1%. Protein identification was performed with at least one unique peptide. Intensity-based absolute quantification (iBAQ) in MaxQuant was performed on the identified peptides to quantify protein abundance in mixture.

### 2.8. Statistical Analysis

Statistical analysis was performed using SPSS version 21.0 and GraphPad Prism (GraphPad Software Inc., La Jolla, CA, USA) software. Population data were provided as median with 95% confidence interval. A Fisher's exact test was used to evaluate differences in terms of age and sex between healthy control subjects and cancer patients, as indicated.

Comparison of EV counts was evaluated by nonparametric Kruskal–Wallis *H* test, as appropriated. Median overall survival (OS) was evaluated using the Kaplan–Meier curve estimator. In survival analysis, events were established as cancer-related death. The logrank test was used to compare median OS. A Cox proportional hazards model was employed to calculate the hazard ratio. The data cutoff was set on February 2019.

The statistical significance was accepted for *p* < 0.05.

Disease control rate (DCR) was used to define responders and nonresponders and relative proportions and ratios according to CD133 + CD326− EV count threshold were evaluated. Cutoff values were generated with the receiving operator characteristic (ROC) curve, and the corresponding area under the curve (AUC) was reported, as indicated. Optimal cutoff values of ROC curves were identified through the Youden index.

## 3. Results

### 3.1. EVs and EV Subtypes in Cancer Patients

Peripheral blood circulating EVs were analysed in patients with advanced cancer and in healthy volunteers; both the total amount of EVs and the concentrations of different EV subpopulations were analysed.

A list of solid tumour immunophenotypical markers was tested according to the literature [49–52]. This list included the following: EpCaM (CD326), CD133, CD90, and CD29. A number of different EV subpopulations were established by combining different markers (CD133 + CD326−, CD133−CD326+, CD133 + CD326+, CD90 + CD29−, CD29 + CD90−, and CD90 + CD29+). Peripheral blood circulating leukocyte-derived (CD45+) and endothelial-derived (CD31+/CD45-/CD41a-) EV levels were also evaluated.

**Figure 1:**
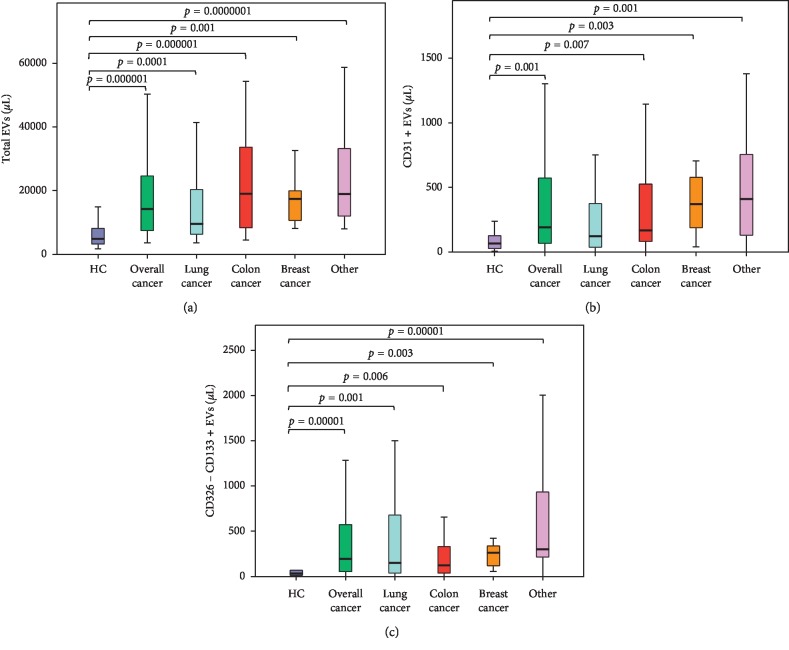
EV concentrations in cancer patients and healthy volunteers. Peripheral blood EV concentrations from healthy subjects and tumour patients (overall, lung, breast, colon, and other tumors) were obtained and analysed. Differences of total EVs (a), CD31+ (b), and CD326−CD133 + EVs (c) between patients and healthy controls (HC) were calculated and reported as box plots. Horizontal black lines represent median values. Statistical comparison was performed by the Kruskal–Wallis H test. Extreme values were not shown.

Confirming previously reported data [53], we demonstrated that the overall blood concentration of EVs resulted significantly higher in cancer patients than in healthy volunteers (cancer patients: median = 14,308 EVs/*μ*l; 95% CI 4,368–70,763; healthy volunteers: median = 5,207 EVs/*μ*l; 95% CI 1751–13531; *p* value = 0.000001). We further stratified the cancer patient population according to the primary tumour site. As shown in Figure 1 and reported in Table 2, higher concentrations of total EVs were detected in all cancer patient groups when paralleled to their age- and gender-matched healthy controls.

We further analysed the differences in EV subtype concentrations between cancer patients and healthy control subjects (Table 2 and Figure 1). Flow cytometry data revealed that cancer patients presented higher concentrations of CD31+ endothelial-derived and CD133 + CD326− tumour cancer stem cell-derived EVs, when compared to healthy volunteers. A cutoff value of CD133 + CD326− EV levels distinguishing cancer patients and healthy controls has been identified (82.5 EVs/*μ*l) with a sensitivity and specificity of 0.69 and 0.84, respectively (Supplementary [Supplementary-material supplementary-material-1]).

Higher concentrations of CD31 + EVs have been identified in breast and colorectal patients, while higher concentrations of CD133 + CD326− EVs have been observed in lung, breast, and colorectal cancer patients. Notably, a ten-fold increase of the concentrations of the epithelial committed cancer stem cell-derived EV subpopulation (CD133+/CD326+) was detected in lung cancer patients, when compared to healthy subjects, although such a subset did not significantly change between all cancer patients and healthy controls. Breast cancer patients also displayed lower levels of CD29 + CD90+/− and CD326 + CD133− than healthy subjects.

### 3.2. Prognostic Role of EVs in Cancer Patients

**Figure 2:**
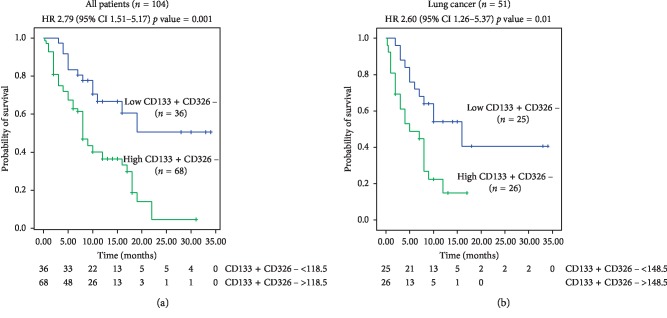
CD326−CD133+ EVs-related survival analysis. (a) The Kaplan–Meier survival curves for the overall cancer population (*n* = 104) were calculated on the basis of the peripheral blood concentrations of CD326-CD133 + EVs. (b) The Kaplan–Meier survival curves for lung cancer patients (*n* = 51) were calculated on the basis of the peripheral blood concentration of CD326-CD133 + EVs.

We then investigated whether EV concentrations could be related to clinical outcomes. Based on the results of the comparative analysis between cancer patients and healthy volunteers, we focused on a possible correlation between total, CD31 +, or CD133 + CD326− EV concentrations and the overall survival of the total cancer patient cohort or of the cancer subgroups (Figure 2). We observed that total EV concentration was not significantly related to differences in overall survival (OS), when all cancer patients were analysed (HR 1.36, 95% CI 0.81–2.30, *p*=0.25). A higher median OS was detected in patients displaying lower peripheral blood EV counts, but such a survival advantage was not statistically significant in the overall cancer population (Supplementary [Supplementary-material supplementary-material-1]). The same findings were obtained when CD31 + EVs were analysed (Supplementary [Supplementary-material supplementary-material-1]). Although patients with a lower number of circulating CD31 + EVs (<120 EVs/*μ*l) presented a higher survival probability, this result was not supported by statistical significance in the overall population (Supplementary [Supplementary-material supplementary-material-1]).

Otherwise, a remarkable and statistically significant difference in OS was detected between the two groups of patients that displayed different concentrations of CD133 + CD326− EVs in the whole cancer population (HR 2.79; 95% CI 1.51–5.17, *p*=0.001; Supplementary [Supplementary-material supplementary-material-1]/Figure 2). The cutoff value between these two groups was 118.5 EVs/*μ*l. Median OS was not reached in the group of patients with low CD133 + CD326− EV counts, compared to a median OS of 8 months for patients with higher concentrations of CD133 + CD326− EVs (Figure 2(a) and Supplementary [Supplementary-material supplementary-material-1]). Of note, no differences in terms of age or gender between these two groups of patients were evidenced (Supplementary [Supplementary-material supplementary-material-1]). Furthermore, we stratified our survival analysis according to the cancer subgroups, focusing our attention on lung and colon cancer patients that resulted in the most copious cohorts in our study population (as shown in Supplementary [Supplementary-material supplementary-material-1]).

We demonstrated that lung cancer patients with high CD133 + CD326− EV concentrations had 2.6 times higher risk of death, compared to those with lower CD133 + CD326− EV peripheral blood concentrations (HR 2.60; 95% CI 1.26–5.37; *p*=0.01; Figure 2(b), Supplementary [Supplementary-material supplementary-material-1]). When we further stratified the patients for the line of therapy, we observed that this advantage was also confirmed in the group of treatment-naïve lung cancer patients (data not shown). No difference in terms of gender and age distribution between the two groups of lung cancer patients was demonstrated (Supplementary [Supplementary-material supplementary-material-1]).

A possible correlation between CD133 + CD326− EV levels and clinical benefit from cancer treatments was explored both in the overall cohort and in lung cancer patients. We observed that almost a 60% of patients who achieved a response or a stable disease after anticancer therapy presented lower circulating EV levels at the baseline (odds ratio 1.83; 95% CI 1.30–13.8; *p*=0.0003; Supplementary [Supplementary-material supplementary-material-1]). Additionally, higher CD133 + CD326− EV concentrations were detected in a large proportion of patients who experienced a progressive disease (78.4%, Supplementary [Supplementary-material supplementary-material-1]). Similar findings were reported for the lung cancer group of patients (Supplementary [Supplementary-material supplementary-material-1]).

As peripheral blood samples were collected at the baseline and at the first disease radiological evaluation, modifications in EV levels during cancer treatment were analysed (Supplementary Tables [Supplementary-material supplementary-material-1] and [Supplementary-material supplementary-material-1]). In particular, we studied how the variations in terms of CD133 + CD326− EV levels were related to the disease status. Interestingly, we observed that decreased EV levels were frequently related to a progressive disease at the time of the first radiologic evaluation (odds ratio 0.33; 95% CI 0.13–0.84; *p*=0.019). Accordingly, the 66.7% of responder patients presented increasing or stable CD133 + CD326− EV levels (odds ratio 1.46; 95% CI 1.04–2.05; *p*=0.019, Supplementary [Supplementary-material supplementary-material-1]).

### 3.3. Analysis of EV Protein Cargo in Lung Cancer Patients

**Figure 3:**
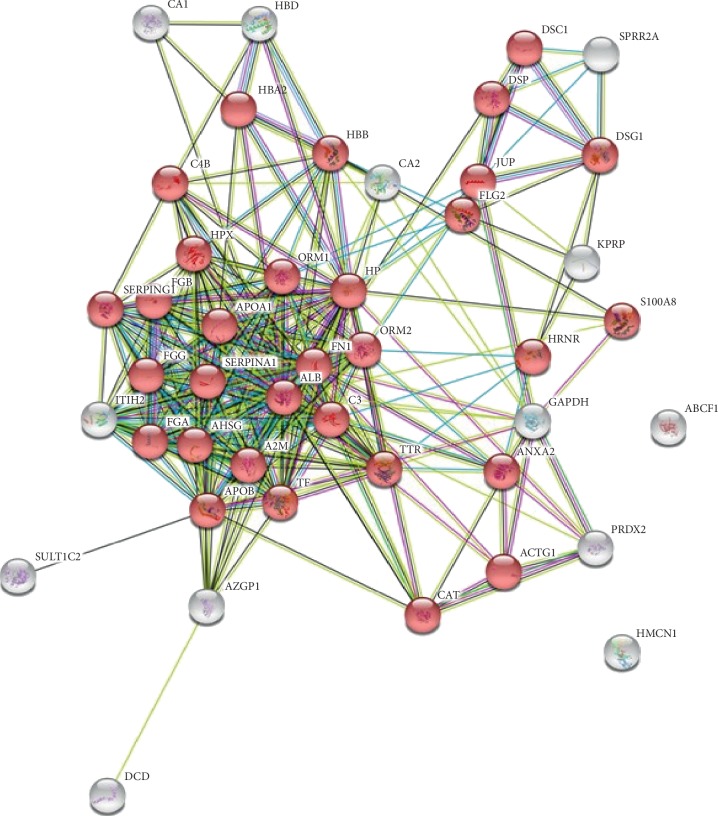
Network of interaction obtained by STRING analysis (https://string-db.org/) of EV-identified proteins. Gene Ontology Classification of proteins was reported. Red dots represent proteins classified as “vesicle-mediated transport” (GO: 0016192).

EV samples from a selected and well-classified cohort of patients affected by lung cancer (*N* = 6) were pulled, and related protein cargoes were analysed and compared to healthy volunteers (*N* = 3). Total and intact EVs were identified as LCD+/phalloidin− events and separated by fluorescence-activated cell sorting. A high level of purity was reached (>90%), and 2.0 × 10 [6] sorted EVs from each condition were analysed in triplicate by a shotgun proteomics approach, as already described [10]. The list of the identified proteins, in at least one replicate for each condition, was reported in Supplementary [Supplementary-material supplementary-material-1] and mainly classified as “vesicle-mediated transport” (GO: 0016192; *p*=1.43*e*^−20^) as reported in Figure 3 (red dots), confirming the efficiency of the used isolation protocol. As reported in Supplementary [Supplementary-material supplementary-material-1], we identified 48 EV proteins in healthy subjects and 42 proteins in EVs from cancer patients (Supplementary [Supplementary-material supplementary-material-1]; Supplementary [Supplementary-material supplementary-material-1]). Interestingly, six proteins were identified only in cancer EVs and three of them resulted from the “cell-cell adhesion” processes, as reported in Supplementary [Supplementary-material supplementary-material-1]; (red dots, *p*=0.0023). Otherwise, twelve proteins, most of them related to the “regulation of peptidase activity” (Supplementary [Supplementary-material supplementary-material-1]; GO: 0052547) process, were identified only in EVs from healthy volunteers.

**Figure 4:**
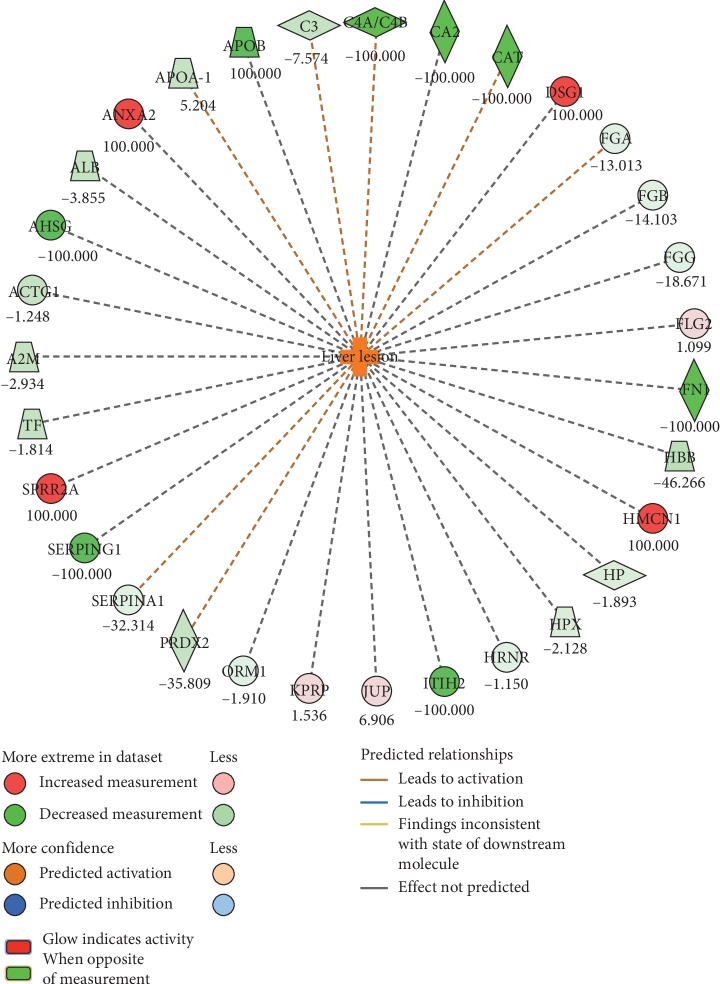
Toxic function evaluation in cancer EVs. The graph represents the Ingenuity Pathway results, providing the related Downstream Regulator analysis of proteins detected in cancer EVs.

Finally, an Ingenuity Pathway analysis (IPA), based on quantitative proteomics data of all identified proteins, was carried out. Data reported in Figure 4 highlight that more than 30 identified proteins allowed the activation of the “Liver Lesion,” as toxic function in cancer EVs (*p*=9.78*E* − 05, z-score = 2.621). The IPA upstream regulator analysis, based on the prior knowledge of expected effects between transcriptional regulators and their target genes [54], was then performed. Results showed that the “zinc finger protein 106,” which is involved in the insulin receptor signalling pathway, was the main activated upstream in cancer (*p* value = 2.9*E* − 06, z-score = 2.0).

## 4. Discussion

Cancer is the second leading cause of death worldwide [55]. New biomarkers are needed to improve cancer diagnosis and the evaluation of patient outcomes [56]. Extracellular vesicles are released by all cell types, reflecting the biological frame of the cellular complexity of each patient [57]. EVs are able to transport specific DNA fragments, RNAs, mi-RNAs, and proteins to target cells. The EV biological content and the physical durability of EVs make them a high suitable and promising material to be employed as a stable and sensitive source of cancer biomarkers [58]. Thus, the characterisation of EV biological complexity may represent a reliable surrogate of the patient pathophysiological status. Few studies have been published on the *ex-vivo* characterisation of peripheral EV subtypes in cancer patients [32–34]. Moreover, all state-of-the-art protocols rely on a number of preanalytical enrichment steps which induce artefact generation. We have developed a simplified flow cytometry method for EV characterisation that does not require any preanalytical enrichment procedure, thus relying on nonmanipulated material and allowing a more reliable picture of the patient condition [7, 10, 59]. Such a method has been applied here to analyse EV concentrations and phenotypes in a large cohort of cancer patients. Interestingly, we demonstrated that cancer patients displayed a significantly higher concentration of peripheral blood circulating EVs. Given that the intercellular crosstalk is particularly active in cancer, this strong exchange of information is probably reflected by high circulating levels of EVs. This finding is in agreement with the recent literature pointing out the pathophysiological role of EVs as cancer hallmarks [57]. In order to dissect the EV involvement in cancer complexity, we analysed different EV subtypes, possibly linked to tumour pathogenesis. In such a context, it was interesting to note that endothelial-derived EVs were significantly increased, in terms of concentration, in cancer and especially in colon cancer patients. It is well known that colon cancer is highly dependent on tumour neovasculogenesis [60], and we plan to enlarge our study cohort of colon cancer patients in order to understand the potential of endothelial EVs as biomarkers to predict or monitor the antiangiogenetic therapy outcomes.

On the other hand, our data strongly suggest a role for CD133 + CD326− EVs in the context of cancer development. As a matter of fact, CD133 was originally identified as a surface antigen of hematopoietic stem cells and as a marker for other embryonic epithelia. Currently, CD133, in association with the lack of CD326 [61], is broadly recognised as a stem cell marker, even though its biological function is still not globally understood [49]. Indeed, EVs deriving from cancer stem cells display the same phenotype of their parental cells [62]. In this context, we demonstrated a statistically significant increase of CD133 + CD326− cancer stem cell-derived EVs in cancer patients (*p*=0.00001). A cutoff value of CD133 + CD326− EV levels distinguishing cancer patients and healthy controls has been identified (82.5 EVs/*μ*l) with high sensitivity and specificity. This means that, if confirmed by further and enlarged studies, circulating CD133 + CD326− EVs could represent a potential useful tool for cancer screening and diagnosis. Moreover, our findings suggest a strong correlation between CD133 + CD326− EV concentrations and patient clinical outcomes. In detail, high levels of cancer stem cell-derived EVs (>118.5 EV/*μ*l) in the whole cancer patient cohort were associated with a poor prognosis, in terms of overall survival. Moreover, CD133 + CD326− EV concentrations were also related to the clinical response rate, given that high levels of this EV subset characterized the majority of patients (78.4%) who did not respond to anticancer therapies. These findings were obtained for the whole cohort of cancer patients and were confirmed when we analysed lung cancer patients which resulted the most represented in our setting. These results were consistent with the poor prognostic role of increased cancer tissue CD133 expression, already described in several studies [63]. This suggests the possible active role of the CD133 antigen in the pathophysiological mechanisms of malignancies, and it strongly supports the idea that the levels of CD133 + EVs circulating in the peripheral blood possibly reflect the complex scenario characterizing the cellular frameworks of the tumour.

As an opening approach for the molecular characterisation of cancer EVs, we carried out a quantitative proteomics analysis in lung cancer EVs, highlighting differential protein expression that, in turn, could be involved in cancer-related biological processes. In particular, Desmoplakin (DSP), Desmocollin-1 (DSC1), Desmoglein-1 (DSG1), and small proline-rich protein 2A (SPRR2A) were identified only in lung cancer EVs and resulted from “cell-cell adhesion” process that, as already described, plays a pivotal role in the development and progression of cancer [64]. Moreover, more than 30 identified proteins allowed the activation of the “Liver Lesion” as a toxic function in cancer EVs. Interestingly, among the pooled and analysed subjects, one patient presented multiple liver metastasis at the time of the observation, while another patient has developed liver metastases several months after blood sample collection. However, further studies in larger cohorts of cancer patients with liver metastasis are needed to understand the biological and clinical implications of this finding.

Altogether, these results demonstrated that EVs could represent a surrogate marker of the tumour complexity, able to capture, in each moment, the development status of the disease and/or the response to treatments.

Therefore, the study of phenotypes, concentrations, and cargoes of the EVs could open a novel view of the liquid biopsy of the future.

## Figures and Tables

**Table 1:** Reagent list-Panel 1-Panel 2-Panel 3.

Reagent	Fluorochrome/reagent	Vendor	Clone	Catalog number	Amount per test
*Panel 1*					
CD133/2	PE	Miltenyi Biotec	293C3	130-113-186	1 *μ*l
EpCAM	PerCP-Cy5.5	BD Biosciences	(EBA-1)	347199	5 *μ*l
CD45	BV510	BD Biosciences	HI30	626266 (custom kit)	5 *μ*l

*Panel 2*					
CD41a	PE	BD Biosciences	HIP8	626266 (custom kit)	5 *μ*l
CD31	PE-Cy7	BD Biosciences	WM59	626266 (custom kit)	5 *μ*l
CD45	BV510	BD Biosciences	HI30	626266 (custom kit)	5 *μ*l

*Panel 3*					
CD90	FITC	BD Biosciences	5E10	555595	1 *μ*l
CD29	PE	BD Biosciences	MAR4	555443	3 *μ*l
CD45	BV510	BD Biosciences	HI30	626266 (custom kit)	5 *μ*l
CD235a	BV421	BD Biosciences	GA-R2 (HIR2)	562938	5 *μ*l

FITC = fluorescein isothiocyanate; PE = R-phycoerythrin; PerPC-Cy5.5 = peridinin-chlorophyll proteins-cyanine 5.5; PE-Cy7 = PE-Cyanine 7, BV = Brilliant Violet.

**Table 2:** Analysis of EV concentrations in cancer patients and healthy volunteers.

	EVs/*μ*l (CI 95%)	*p* value
*Total EV*		
Controls	5207 (1751–13531)	
Cancer	14308 (4368–70763)	**0.000001**
Lung cancer	9600 (3867–75021)	**0.0001**
Colorectal cancer	19044 (5257–1745393)	**0.000001**
Breast cancer	17437 (8079-NE)	**0.001**
Other	19012 (8047-NE)	**0.0000001**

*CD31+*		
Controls	70 (7–268)	
Cancer	168 (0.5–1297)	**0.001**
Lung cancer	123 (5–1021)	0.058
Colorectal cancer	168 (1–2826)	**0.007**
Breast cancer	371 (39-NE)	**0.003**
Other	411 (0-NE)	**0.001**

*CD90-CD29+*		
Controls	150 (11–1573)	
Cancer	168 (1–2924)	0.668
Lung cancer	297 (10–2467)	0.165
Colorectal cancer	279 (0–4347)	0.515
Breast cancer	39 (23-NE)	**0.02**
Other	83 (0-NE)	0.273

*CD326-CD133+*		
Controls	34 (0–260)	
Cancer	194 (0–2286)	**0.00001**
Lung cancer	151 (4–3376)	**0.001**
Colorectal cancer	123 (0–2827)	**0.006**
Breast cancer	262 (55-NE)	**0.003**
Other	300 (0-NE)	**0.00001**

*CD326* *+* *CD133-*		
Controls	742 (20–2545)	
Cancer	554 (15–2546)	0.155
Lung cancer	650 (50–2188)	0.476
Colorectal cancer	899 (5–7678)	0.775
Breast cancer	150 (10-NE)	**0.003**
Other	177 (0-NE)	**0.015**

*LEUKO-EV*		
Controls	238 (37–1721)	
Cancer	265 (42–1351)	0.529
Lung cancer	328 (34–1680)	0.086
Colorectal cancer	274 (47–2416)	0.522
Breast cancer	66 (38-NE)	**0.036**
Other	182 (64-NE)	0.407

*CD90* *+* *CD29-*		
Controls	280 (24–3341)	
Cancer	143 (6–5606)	0.161
Lung cancer	62 (0–2276)	0.058
Colorectal cancer	145 (8.2–17356)	0.437
Breast cancer	193 (24-NE)	0.342
Other	182 (17-NE)	0.980

*CD90* *+* *CD29+*		
Controls	134 (11–571)	
Cancer	84 (0–615)	0.110
Lung cancer	87 (0–675)	0.232
Colorectal cancer	109 (7–788)	0.543
Breast cancer	9 (0-NE)	**0.001**
Other	82 (6-NE)	0.160

*CD326* *+* *CD133+*		
Controls	17 (0–83)	
Cancer	63 (0–739)	0.124
Lung cancer	172 (0–1312)	**0.002**
Colorectal cancer	87 (0–2670)	0.204
Breast cancer	0 (0-NE)	**0.002**
Other	5 (0-NE)	0.435

## Data Availability

The scientific data used to support the findings of this study are included within the article and the supplementary information file.

## Supplementary Materials

Supplementary Materials 1 Supplementary Table 1: patient characteristics. Supplementary Table 2: survival analysis. Supplementary Table 3: univariate analysis of CD133 + CD326− EVs. Supplementary Table 4: clinical benefit rate evaluated according to CD133 + CD326-EV concentrations. Supplementary Table 5: baseline EV concentrations in cancer patients. Supplementary Table 6: EV concentrations in cancer patients at the first radiological evaluation. Supplementary Table 7: variation of CD133 + CD326− EV levels respect to the baseline concentration, evaluated according to the clinical benefit response. Supplementary Table 8: list of proteins identified in EV isolated from healthy control subjects (8A) and from lung cancer patients (8B). Supplementary Figure 1 Panel 1: (A) A region (“platelet-free area”) under the one in which platelets (PLTs) fall was drawn on a SSCH/FSC-H dot-plot. (B) The “platelet-free area” events were then represented on an LCD-H/Phalloidin-H dotplot and EVs were identified as LCD-positive/phalloidin-negative dots. (C) EVs (LCD+/phalloidin− events) were analysed on a CD45-H/CD133-H dotplot and CD45 + events were gated; a CD45-negative logical gate was then set, and the resulting population was plotted on a (D) CD326-H/CD133-H dotplot, where CD133+/CD326−, CD133+/CD326+, and CD133−/CD326 + EVs were identified. Panel 2: (E) EVs identified as shown in A and B were represented on a CD31-H/CD41a-H dotplot, and events showing the CD31+/CD41a + phenotype were identified as platelet-derived EVs (PLT-EVs); a PLT-EVs-negative logical gate was set. (F) The PLT-EV-negative population was plotted on a CD45-H/CD31-H dotplot, and CD45 + events were identified as leukocyte-derived EVs, while the CD31+/CD45− compartment was defined as the endothelialderived EV population. Panel 3. (G) The “platelet-free area” events were identified as described in A and then represented on an LCD-H/CD235a-H dotplot; EVs were identified as LCD-positive/CD235a-negative dots. (H) Those events were analysed on a CD45-H/CD90-H dotplot, and CD45 + events were gated. A CD45-negative logical gate was set. (I) The resulting population was plotted on a CD29-H/CD90-H dotplot, where CD90+/CD29−, CD90+/CD29+, and CD90−/CD29 + EVs were identified. Supplementary Figure 2: ROC curves were calculated to determine the power of total EV (a), CD31 + EV (b), and CD133 + CD326− EV (c) concentrations as a discriminator of patients and healthy volunteers. Supplementary Figure 3: (A) Venn diagram of the identified proteins in healthy control (HC) pooled EVs and in lung cancer polled EVs. (B) Three of the six proteins (reported as red dots) identified only in cancer EVs resulted from the “cell-cell adhesion” process (*p*=0.0023). (C) Ten proteins identified only in EVs from healthy volunteers resulted from the “regulation of peptidase activity” (GO:0052547).
