# Corrigendum to “Circulating Cancer Stem Cell-Derived Extracellular Vesicles as a Novel Biomarker for Clinical Outcome Evaluation”

**DOI:** 10.1155/2020/8947367

**Published:** 2020-03-31

**Authors:** D. Brocco, P. Lanuti, P. Simeone, G. Bologna, D. Pieragostino, M. C. Cufaro, V. Graziano, M. Peri, P. Di Marino, M. De Tursi, A. Grassadonia, I. G. Rapposelli, L. Pierdomenico, E. Ercolino, F. Ciccocioppo, P. Del Boccio, M. Marchisio, C. Natoli, S. Miscia, N. Tinari

**Affiliations:** ^1^Clinical Oncology Unit, SS Annunziata Hospital, Chieti, Italy; ^2^Department of Medicine and Aging Sciences, University “G. D'Annunzio” of Chieti-Pescara, Chieti, Italy; ^3^Centre on Aging Sciences and Translational Medicine (Ce. S. I.-Me. T.), University “G. D'Annunzio” of Chieti- Pescara, Chieti, Italy; ^4^Department of Medical, Oral and Biotechnological Sciences, University “G. D'Annunzio” of Chieti-Pescara, Analytical Biochemistry and Proteomics Laboratory, Chieti, Italy; ^5^Cancer Research UK Cambridge Institute, University of Cambridge, Cambridge CB2 0RE, UK; ^6^Department of Medical, Oral and Biotechnological Sciences, Gabriele D'Annunzio University, Chieti, Italy; ^7^Department of Medical Oncology, Istituto Scientifico Romagnolo per lo Studio e la Cura dei Tumori (IRST) IRCCS, Meldola, Italy; ^8^Department of Pharmacy, University “G. D'Annunzio” of Chieti-Pescara, Chieti, Italy

In the article titled “Circulating Cancer Stem Cell-Derived Extracellular Vesicles as a Novel Biomarker for Clinical Outcome Evaluation” [1], the author Piero del Boccio was affiliated to Department of Medical, Oral and Biotechnological Sciences, University “G. D'Annunzio” of Chieti-Pescara, Analytical Biochemistry and Proteomics Laboratory, Chieti, Italy, which is incorrect. The corrected list of affiliations is shown above.
