# Supplementary material for: Circulating Cancer Stem Cell-Derived Extracellular Vesicles as a Novel Biomarker for Clinical Outcome Evaluation
Source: J Oncol. 2019 Nov 18;2019:5879616. doi: 10.1155/2019/5879616 (PMC6885781; doi:10.1155/2019/5879616)
Supplement: Supplementary Materials 1 — Supplementary Table 1: patient characteristics. Supplementary Table 2: survival analysis. Supplementary Table 3: univariate analysis of CD133 + CD326− EVs. Supplementary Table 4: clinical benefit rate evaluated according to CD133 + CD326-EV concentrations. Supplementary Table 5: baseline EV concentrations in cancer patients. Supplementary Table 6: EV concentrations in cancer patients at the first radiological evaluation. Supplementary Table 7: variation of CD133 + CD326− EV levels respect to the baseline concentration, evaluated according to the clinical benefit response. Supplementary Table 8: list of proteins identified in EV isolated from healthy control subjects (8A) and from lung cancer patients (8B). Supplementary Figure 1 Panel 1: (A) A region (“platelet-free area”) under the one in which platelets (PLTs) fall was drawn on a SSCH/FSC-H dot-plot. (B) The “platelet-free area” events were then represented on an LCD-H/Phalloidin-H dotplot and EVs were identified as LCD-positive/phalloidin-negative dots. (C) EVs (LCD+/phalloidin− events) were analysed on a CD45-H/CD133-H dotplot and CD45 + events were gated; a CD45-negative logical gate was then set, and the resulting population was plotted on a (D) CD326-H/CD133-H dotplot, where CD133+/CD326−, CD133+/CD326+, and CD133−/CD326 + EVs were identified. Panel 2: (E) EVs identified as shown in A and B were represented on a CD31-H/CD41a-H dotplot, and events showing the CD31+/CD41a + phenotype were identified as platelet-derived EVs (PLT-EVs); a PLT-EVs-negative logical gate was set. (F) The PLT-EV-negative population was plotted on a CD45-H/CD31-H dotplot, and CD45 + events were identified as leukocyte-derived EVs, while the CD31+/CD45− compartment was defined as the endothelialderived EV population. Panel 3. (G) The “platelet-free area” events were identified as described in A and then represented on an LCD-H/CD235a-H dotplot; EVs were identified as LCD-positive/CD235a-negative dots. (H) Those events were analysed on a [file 5879616.f1.pdf]

Supplementary Table 1. Patient characteristics

Variable	Cancer Patients		Healthy Controls		p-value (Fisher's Exact Test)
	Number (n=106)	Percentage	Number (n=25)	Percentage	
**Age**					
**>65**	63	59.4	13	52	0.51
**≤65**	43	40.6	12	48	
**Sex**					
**Male**	73	68.9	13	52	0.16
**Female**	33	31.3	12	48	
**Primary Cancer Site**					
**Lung**	51	48.1			
**Colon/Rectum**	30	28.3			
**Breast**	7	6.6			
**Other***	18	17.0			
**Cancer Treatment**					
**Chemotherapy**	50	47.2			
**Immunotherapy**	22	20.8			
**Anti-angiogenetic therapy**	19	17.9			
**Anti-EGFR therapy**	9	8.5			
**Hormonal therapy**	3	2.8			
**Other**	3	2.8			
**Line of therapy**					
**1**	69	65.1			
**2**	26	24.5			
**≥3**	11	10.3			

* This group included 4 patients affected by melanoma, 4 patients affected by bladder cancer, 3 patients affected by kidney cancer, 2 patients with pancreatic adenocarcinoma, 2 patients with ovarian cancer, 1 patient affected by hepatocellular carcinoma and 1 patient affected by cholangio-carcinoma.

Supplementary Table 2. Survival analysis.

Parameter	Patients	Cut off (ROC curves)	Hazard ratio	Median OS	p-value
**Age**	106		1.00 (0.98-1.03)		0.70
**Sex**	106		0.37 (0.19-0.68)		**0.002**
**Total EVs**	106		1.36 (0.81-2.30)		0.25
		<11886		16.0 (NE-NE)	0.23
		>11886		10.0 (3.5-16.5)	
**CD31+EVs**	81		1.55 (0.83-2.88)		0.16
		<120		19.0 (5.0-33.0)	0.15
		>120		10.0 (6.3-13.7)	
**CD133+CD326-EVs**	104		2.79 (1.51-5.17)		**0.001**
		<118.5		NR (3.0-NE)	**0.0004**
		>118.5		8.0 (6.6-9.4)	
**Lung Cancer**	51		2.60 (1.26-5.37)		**0.01**
		<148.5		16.0 (6.6-25.4)	**0.006**
		>148.5		5.0 (0.2-9.8)	
**Colon Cancer**	29		2.61 (0.72-9.42)		0.14
		<78.5		19.0 (NE-NE)	0.11
		>78.5		10.0 (4.1-16.0)	

Supplementary Table 3. Univariate Analysis of CD133+CD326- EVs.

Parameter	Threshold	Patients	Median (CI 95%)	p-value
**Total cancer patient cohort**				
**Age**				0.761
	<118.5	68	68.0 (45.4-82.1)	
	>118.5	36	68.0 (47.9-81.6)	
**Sex**				1.000
**male**	<118.5	51		
**female**		17		
**male**	>118.5	27		
**female**		9		
**Lung Cancer**				
**Age**				0.797
	<148.5	33	70.0 (48.0-81.0)	
	>148.5	18	69.5 (42.4.-80.3)	
**Sex**				0.916
**male**	<148.5	29		
**female**		4		
**male**	>148.5	16		
**female**		2		

Supplementary Table 4. Clinical benefit rate evaluated according to CD133+CD326-EV concentrations.

Group	Threshold	Percentage %	Odd Ratio (CI 95%)	p-value
**Total cancer patient cohort**				
**Responders (n=57)**	<148.5	59.6 (n=34)	1.83 (1.30-13.8)	**0.0003**
	>148.5	40.4 (n=23)		
**Non-Responders (n=37)**	<148.5	21.6 (n=8)	0.34 (0.18-0.67)	**0.0003**
	>148.5	78.4 (n=29)		
**Lung Cancer**				
**Responders (n=26)**	<148.5	69.2 (n=18)	2.44 (2.08-28.0)	**0.002**
	>148.5	30.8 (n=8)		
**Non-Responders (n=22)**	<148.5	22.7 (n=5)	0.32 (0.14-0.73)	**0.002**
	>148.5	77.3 (n=17)		

Supplementary Table 5. Baseline EV concentrations in cancer patients.

Patients	Total EVs	Leuko EVs	CD31+ EVs	CD90+CD29- EVs	CD90-CD29+ EVs	CD90+CD29+ EVs	CD326-CD133+ EVs	CD326+CD133+ EVs	CD326+CD133- EVs
1	11702,2	63,9	118,7	28,0	25,2	5,6	60,4	2,7	82,4
2	33216,8	607,9	1377,9	10775,8	12,7	496,4	1449,7	13,5	171,6
3	6650,0	NE	NE	43,4	347,2	23,8	37,8	224,0	896,0
4	12030,9	112,6	1183,9	142,9	52,5	35,0	283,0	0,0	252,6
5	9600,4	41,7	163,8	457,7	16,2	43,1	373,3	7,8	129,6
6	101960,6	NE	NE	42,0	259,0	91,0	623,0	716,8	140,0
7	34086,8	458,2	585,5	320,8	11,7	11,7	183,9	2,7	0,0
8	18727,9	75,8	166,3	126,0	129,5	49,0	194,9	68,6	592,9
9	15421,3	158,3	313,5	163,8	100,4	39,6	215,2	0,0	18,1
10	16744,0	630,0	70,0	1499,4	16,8	215,6	123,2	268,8	554,4
11	18282,0	276,6	225,4	5870,8	0,3	0,0	0,0	0,0	10,2
12	9193,8	655,2	5,2	728,0	3057,6	196,0	26,6	201,6	569,8
13	21148,4	103,6	526,4	4104,8	36,4	100,8	271,3	0,0	26,3
14	7149,8	NE	NE	0,0	1358,0	404,6	51,8	191,8	1134,0
15	4253,2	NE	NE	29,4	560,0	154,0	14,0	197,4	695,8
16	16573,2	264,6	93,8	26,6	263,2	86,8	16,8	0,0	49,0
17	3754032,8	789,6	56,0	2771,2	229,6	324,8	655,2	95,2	929,6
18	8047,2	589,4	215,6	590,8	0,0	61,6	492,8	310,8	642,6
19	10838,8	134,4	873,6	16,8	543,2	105,0	303,8	0,0	3634,4
20	14359,8	NE	NE	1,0	40,6	44,6	42,0	23,8	749,0
21	18877,1	168,6	751,4	57,1	37,1	2,9	58,0	2,0	107,0
22	13312,4	59,1	388,9	1974,9	17,9	110,2	216,0	3,0	42,0
23	41397,5	327,5	235,0	45,0	57,5	0,0	266,5	0,0	51,2
24	12789,8	127,6	102,7	33,6	33,6	8,4	172,9	2,7	189,4
25	21136,9	130,7	1216,4	211,3	74,0	39,6	398,5	0,0	32,3
26	21927,2	44,9	118,9	208,7	73,8	22,9	221,1	0,0	29,5
27	17210,2	NE	NE	32,2	1239,0	21,0	2630,6	495,6	2039,8
28	58753,3	1320,7	634,7	538,8	59,4	182,4	2003,8	26,3	555,6
29	20706,3	93,3	1301,2	145,3	105,7	13,2	NE	NE	NE
30	54311,3	55,4	58,3	83,5	70,0	18,8	0,0	NE	22,4
31	23470,0	723,3	1970,0	140,0	0,0	524,0	776,0	8,0	228,0
32	21040,6	185,2	1142,6	144,4	564,4	13,1	61,3	NE	30,6
33	27163,8	298,9	132,4	186,7	66,1	31,1	383,0	5,0	52,0
34	8534,4	184,8	250,6	700,0	600,6	484,4	295,4	470,4	1265,6
35	35923,1	735,0	21,9	173,9	72,1	135,8	704,4	91,9	411,3
36	37175,7	889,2	128,6	339,4	933,3	29,7	1282,6	18,1	180,6
37	50277,0	1098,8	509,1	1982,6	0,0	361,3	1255,5	4,5	121,9
38	66981,3	748,1	538,1	406,9	4,4	888,1	903,0	39,0	134,0
39	33642,8	2768,9	462,8	268,0	128,0	132,0	139,0	7,0	71,0
40	5880,0	NE	NE	32,2	298,2	18,2	95,2	200,2	1075,2
41	10476,2	NE	NE	23,8	2384,2	110,6	96,6	743,4	1790,6
42	35184,0	1004,0	168,0	20029,0	4,5	835,5	1625,8	13,5	171,6
43	72800,0	103,6	0,0	50,4	442,4	145,6	0,0	0,0	0,0
44	6300,0	114,8	95,2	5,6	450,8	176,4	29,4	0,0	1831,2
45	4474,4	371,0	712,6	42,0	565,6	225,4	0,0	0,0	670,6
46	41848,8	183,4	0,0	15169,0	70,0	452,2	329,0	371,0	991,2
47	8414,0	NE	NE	61,6	3950,8	61,6	1498,0	182,0	947,8
48	4495,4	NE	NE	9,8	63,0	116,2	0,0	78,4	586,6
49	24623,2	737,8	140,0	1125,6	420,0	103,6	308,0	453,6	1069,6
50	6445,6	NE	NE	32,2	379,4	18,2	151,2	537,6	1372,0
51	8423,8	274,4	155,4	15,4	320,6	15,4	100,8	4,2	1463,0
52	11060,0	70,0	218,4	2879,8	298,2	359,8	830,2	410,2	1162,0
53	4998,0	NE	NE	37,8	14,0	228,2	140,0	0,0	259,0
54	6456,8	NE	NE	16,8	673,4	53,2	119,0	361,2	618,8
55	37440,2	NE	NE	1087,8	345,8	0,0	28975,8	1642,2	798,0
56	7502,6	NE	NE	173,6	82,6	116,2	47,6	302,4	963,2
57	87080,0	NE	NE	471,8	42,0	0,0	1734,6	466,2	649,6
58	61833,8	NE	NE	110,6	148,4	50,4	721,0	292,6	1433,6
59	20808,2	NE	NE	36,4	358,4	67,2	1793,4	170,8	1114,4
60	8575,0	2452,8	974,4	274,4	168,0	39,2	565,6	536,2	156,8
61	7229,6	NE	NE	44,8	394,8	60,2	19,6	324,8	974,4
62	15773,8	NE	NE	0,0	3621,8	653,8	130,2	134,4	2006,2

63	6458,2	NE	NE	64,4	194,6	215,6	54,6	99,4	1521,8
64	7593,6	NE	NE	37,8	42,0	228,2	187,6	266,0	246,4
65	4054,4	NE	NE	5,6	1696,8	78,4	5,6	301,0	393,4
66	12507,0	204,4	116,2	128,8	1271,2	75,6	0,0	0,0	3309,6
67	6647,2	NE	NE	28,0	401,8	42,0	0,0	67,2	666,4
68	5938,8	151,2	26,6	16671,2	75,6	107,8	893,2	177,8	1519,0
69	4746,0	840,0	750,4	225,4	131,6	49,0	32,2	23,8	64,4
70	4312,0	179,2	28,0	256,2	597,8	84,0	126,0	65,8	60,2
71	6531,0	145,6	162,4	425,6	1276,8	749,0	441,0	312,2	898,8
72	34251,0	140,0	123,2	1873,2	93,8	315,0	140,0	529,2	383,6
73	6227,2	149,8	429,8	1031,8	1384,6	368,2	576,8	602,0	1047,2
74	33233,2	1486,8	5,6	487,2	358,4	28,0	315,0	280,0	810,6
75	14453,6	308,0	308,0	1528,8	140,0	390,6	140,0	380,8	453,6
76	3586,8	190,4	1209,6	1516,2	92,4	169,4	32,2	0,0	856,8
77	7683,2	78,4	67,2	403,2	296,8	705,6	145,6	224,0	515,2
78	4911,2	403,2	560,0	588,0	151,2	33,6	28,0	358,4	291,2
79	30794,4	945,0	239,4	33,6	1629,6	106,4	1860,6	0,0	359,8
80	6944,0	0,0	0,0	39,2	324,8	112,0	823,2	50,4	929,6
81	6036,8	151,2	16,8	16,8	145,6	78,4	49,0	0,0	43,4
82	6218,8	303,8	145,6	40,6	1173,2	116,2	653,8	245,0	2993,2
83	7044,8	358,4	36,4	70,0	350,0	70,0	28,0	0,0	89,6
84	12303,2	369,6	11,2	28,0	644,0	28,0	11,2	0,0	296,8
85	9434,6	800,8	39,2	218,4	280,0	151,2	5,6	0,0	127,4
86	21029,4	235,2	3040,8	18,2	5922,0	256,2	123,2	0,0	1276,8
87	19807,2	341,6	16,8	58,8	2613,8	70,0	61,6	5,6	12045,6
88	3585,4	383,6	67,2	53,2	840,0	204,4	25,2	0,0	830,2
89	12677,0	816,2	19,6	0,0	960,4	0,0	4494,0	2786,0	477,4
90	36563,8	473,2	390,6	11,2	1426,6	107,8	3859,8	4267,2	2623,6
91	14256,2	431,2	985,6	543,2	1370,6	180,6	263,2	309,4	980,0
92	21897,4	390,6	46,2	474,6	292,6	103,6	159,6	63,0	2310,0
93	434840,0	851,2	655,2	60,2	1010,8	231,0	226,8	172,2	1183,0
94	40152,0	583,8	72,8	72,8	609,0	189,0	2380,0	721,0	2107,0
95	14017,0	269,0	38,0	22,0	0,0	17,0	22,0	1092,0	1014,0
96	21056,0	140,0	39,2	NE	NE	NE	NE	NE	NE
97	17340,4	NE	NE	176,4	5362,0	228,2	932,4	67,2	1192,8
98	17936,8	1353,8	0,0	4986,8	116,2	110,6	296,8	215,6	554,4
99	13397,1	54,3	320,0	706,8	20,5	17,1	448,0	2,8	274,4
100	18900,0	106,4	75,6	91,8	23,0	9,2	55,0	0,0	15,0
101	17436,7	63,3	1260,0	336,0	91,0	37,3	330,6	0,0	181,7
102	19297,1	205,7	617,1	177,0	92,5	26,4	265,7	2,9	60,0
103	32660,4	130,7	706,2	105,0	55,4	8,8	336,0	0,0	158,7
104	8079,2	37,9	300,4	282,5	44,2	0,0	118,2	0,0	140,0
105	9126,3	40,8	449,2	23,8	32,8	0,0	422,9	0,0	331,4
106	12268,6	65,7	371,4	334,4	28,5	13,0	194,4	2,6	10,4

Supplementary Table 6. EV concentrations in cancer patients at the first radiological evaluation.

Patients2	Total EVs	Leuko EVs	CD31+ EVs	CD90+CD29- EVs	CD90-CD29+ EVs	CD90+CD29+ EVs	CD326-CD133+ EVs	CD326+CD133+ EVs	CD326+CD133- EVs
1	24717,4	157,9	26,8	154,4	14,4	79,0	22,1	3,7	114,2
2	5126,8	123,2	33,6	78,4	173,6	22,4	58,8	198,8	180,6
3	41047,1	609,7	1336,8	325,2	194,2	58,7	1919,4	9,0	555,5
4	17288,5	143,0	190,6	243,9	45,2	45,2	2235,6	7,8	4,4
5	5185,6	186,2	595,0	243,6	1418,2	956,2	91,0	443,8	938,0
6	98744,0	452,0	88,0	261,9	1946,5	207,7	7225,8	31,6	672,9
7	41815,5	432,7	591,8	171,5	49,0	101,5	55,3	0,0	582,1
8	21807,3	592,7	65,3	318,6	67,6	111,0	1306,7	46,7	182,0
9	10147,2	604,8	39,2	16,8	420,0	134,4	0,0	0,0	677,6
10	27174,0	667,3	163,3	14161,9	4,4	183,8	1630,6	16,5	123,5
11	8545,6	560,0	35,0	21,0	1047,2	212,8	50,4	0,0	3354,4
12	12880,0	285,6	0,0	2391,2	649,6	252,0	61,6	1220,8	268,8
13	13756,4	306,6	289,8	317,8	123,2	32,2	60,2	292,6	371,0
14	11447,8	420,0	11,2	9,8	9825,2	29,4	5013,4	1443,4	291,2
15	12180,0	106,4	429,8	407,4	943,6	407,4	127,4	5,6	116,2
16	736442,0	851,2	655,2	60,2	1010,8	231,0	226,8	172,2	1183,0
17	9301,6	168,0	218,4	207,2	39,2	162,4	655,2	196,0	285,6
18	47050,8	236,9	825,6	228,0	160,0	68,0	8,0	0,0	49,0
19	20972,9	731,6	271,0	550,7	42,0	163,3	975,6	17,5	218,8
20	28446,3	161,9	83,1	189,7	45,2	58,7	709,0	0,0	144,5
21	40233,5	86,5	131,8	416,0	44,0	60,0	1111,5	8,5	343,6
22	7222,8	197,2	337,2	177,3	37,3	84,0	1502,9	8,2	366,5
23	4155,2	NE	NE	26,6	302,4	103,6	114,8	225,4	1030,4
24	41339,4	805,0	35,0	305,5	17,0	67,9	6549,0	0,0	0,0
25	60092,0	2216,0	232,0	313,9	178,2	63,6	2068,0	32,0	476,0
26	23788,0	156,0	244,0	272,0	29100,0	16,0	8,2	0,0	490,0
27	41499,4	182,4	207,9	120,6	46,7	38,9	582,6	13,5	329,7
28	18590,6	893,2	75,6	589,4	600,6	683,2	506,8	245,0	576,8
29	46592,9	1174,2	803,9	1045,6	4,4	266,9	1264,5	0,0	4,5
30	24717,4	157,9	801,3	154,4	14,4	79,0	22,1	3,7	114,2
31	40018,5	574,4	499,0	18375,0	4,4	577,5	250,0	4,0	36,0
32	9770,6	NE	NE	518,0	341,6	170,8	581,0	453,6	674,8
33	72461,2	2100,0	134,4	95,2	5936,0	117,6	21589,4	13008,8	2391,2
34	6666,8	247,8	159,6	12,6	505,4	182,0	0,0	0,0	1050,0
35	6440,0	736,4	23,8	54,6	554,4	140,0	0,0	0,0	408,8
36	20367,2	NE	NE	29,4	299,6	130,2	0,0	0,0	2374,4
37	2598,4	403,2	110,6	823,2	392,0	1349,6	56,0	100,8	313,6
38	16234,4	263,2	70,0	1540,0	1449,0	533,4	571,2	386,4	756,0
39	10260,6	159,6	379,4	497,0	2389,8	439,6	824,6	375,2	3925,6
40	8033,2	NE	NE	263,2	259,0	215,6	172,2	189,0	904,4
41	10438,4	0,0	70,0	791,0	33,6	12,6	568,4	49,0	511,0
42	25293,8	649,6	74,2	275,8	1429,4	110,6	109,2	606,2	1518,8
43	8703,8	1450,4	935,2	459,2	784,0	179,2	968,8	600,6	333,2
44	4799,2	350,0	320,6	793,8	665,0	1365,0	280,0	184,8	403,2
45	47320,0	0,0	4060,0	15,4	259,0	358,4	950,6	810,6	1094,8
46	243019,0	233,8	2205,0	344,4	2864,4	256,2	53,2	75,6	18652,2
47	7746,2	280,0	296,8	347,2	173,6	44,8	16,8	373,8	694,4
48	7070,0	89,6	50,4	487,2	197,4	498,4	576,8	198,8	548,8
49	15009,4	660,8	436,8	506,8	490,0	86,8	151,2	326,2	198,8
50	7576,8	175,0	29,0	576,8	274,4	326,2	99,4	274,4	105,0
51	4431,0	184,8	750,4	1058,4	1540,0	683,2	37,8	183,4	431,2
52	24262,0	520,8	33,6	75,6	1416,8	145,6	0,0	0,0	6890,8
53	7247,8	638,4	5,6	2424,8	1467,2	128,0	16,8	312,2	802,2
54	9850,4	840,0	140,0	85,4	1162,0	152,6	70,0	0,0	1820,0
55	4540,2	89,6	33,6	4,2	603,4	82,6	0,0	0,0	670,6
56	5758,2	268,8	70,0	46,2	2660,0	256,2	49,0	5,6	1460,2
57	47107,2	352,8	403,2	40,6	2088,8	75,6	1029,0	12,6	2361,8
58	14915,6	543,2	123,2	26,6	543,2	102,2	5,6	0,0	1631,0
59	97879,6	163,8	70,0	23,8	912,8	182,0	1800,4	4,2	5044,2
60	25695,6	723,8	23,8	49,0	361,2	210,0	134,4	301,0	1729,0
61	35302,4	767,2	95,2	70,0	2864,4	70,0	0,0	5,6	9514,4
62	67736,2	845,6	21,0	130,2	170,8	98,0	21425,6	8680,0	5331,2

63	39991,0	655,2	102,2	207,2	389,2	378,0	2366,0	623,0	2219,0
64	29448,1	297,5	350,0	461,2	98,8	127,6	3593,3	17,0	2494,5
65	59416,6	229,4	95,3	437,0	17,0	114,5	5171,3	35,0	2340,6

**Supplementary Table 7. Variation of CD133+CD326- EV levels respect to the baseline concentration, evaluated according to the clinical benefit response.**				
**Group**	**Difference**	**Percentage %**	**Odd Ratio (CI 95%)**	**p-value (Fisher's exact test)**
**Total cancer patient cohort**				
**Responders (n=48)**	≥ 0	66.7 (n=32)	1.46 (1.04-2.05)	**0.019**
	< 0	33.3 (n=16)		
**Non-Responders (n=16)**	≥ 0	31.3 (n=5)	0.33 (0.13-0.84)	**0.019**
	< 0	68.8 (n=11)		
**Lung Cancer**				
**Responders (n=20)**	≥ 0	65.0 (n=13)	1.52 (0.70-15.1)	0.15
	< 0	35.0 (n=7)		
**Non-Responders (n=11)**	≥ 0	36.4 (n=4)	0.47 (0.17-1.29)	0.15
	< 0	63.6 (n=7)		

Supplementary Table 8. List of proteins identified in EV isolated from Healthy control subjects (8A) and from Lung cancer patients (8B).

Supplementary Table 8A

Healthy Control							
**Protein ID**	**#Peptides**	**#Unique peptides**	**Sequence coverage [%]**	**PTM**	**Mol. weight (kDa)**	**Score**	**Description**
A0A0B4J2B5	1	1	19.4	Carbamidomethylation	10.657	9.3532	Immunoglobulin heavy variable 3/OR16
A6NMB1	1	1	1.5	Carbamidomethylation	52.991	7.26	Sialic acid-binding Ig-like lectin 16
O00338	1	1	4.1	Carbamidomethylation	34.88	6.5525	Sulfotransferase 1C2
P00738	10	10	27.3	Carbamidomethylation, Oxidation (M)	45.205	126.08	Haptoglobin
P00915	6	6	32.6	Carbamidomethylation	28.87	37.302	Carbonic anhydrase 1
P00918	3	3	11.5	Carbamidomethylation	29.246	18.331	Carbonic anhydrase 2
P01009	5	5	12.9	Carbamidomethylation	46.736	31.917	Alpha-1-antitrypsin
P01023	18	18	14	Carbamidomethylation	163.29	125.89	Alpha-2-macroglobulin
P01024	21	21	15.4	Carbamidomethylation	187.15	178.13	Complement C3
P01834	2	2	34.6	Carbamidomethylation	11.765	18.095	Ig kappa chain C region
P01857	8	3	28.8	Carbamidomethylation	36.105	25.922	Ig gamma-1 chain C region
P01860	9	2	24.1	Carbamidomethylation	41.287	61.822	Ig gamma-3 chain C region
P01871	7	7	18.3	Carbamidomethylation	49.439	58.845	Ig mu chain C region
P01876	3	3	8.8	Carbamidomethylation	37.654	20.735	Ig alpha-1 chain C region
P02042	6	2	38.1	Carbamidomethylation	16.055	31.214	Hemoglobin subunit delta
P02647	7	7	29.2	Carbamidomethylation, Oxidation (M)	30.777	52.296	Apolipoprotein A-I
P02671	11	11	15.2	Carbamidomethylation	94.972	72.975	Fibrinogen alpha chain
P02675	10	10	27.9	Carbamidomethylation	55.928	82.572	Fibrinogen beta chain
P02679	6	6	14.3	Carbamidomethylation	51.511	40.592	Fibrinogen gamma chain
P02751	1	1	0.5	Carbamidomethylation	262.62	7.1743	Fibronectin
P02763	3	2	15.9	Carbamidomethylation	23.511	18.42	Alpha-1-acid glycoprotein 1
P02765	2	2	6.5	Carbamidomethylation	39.34	34.259	Alpha-2-HS-glycoprotein
P02766	1	1	8.8	Carbamidomethylation	15.887	6.5995	Transthyretin
P02768	40	35	53	Carbamidomethylation, Oxidation (M)	69.366	323.31	Serum albumin
P02787	24	24	35.7	Carbamidomethylation, Oxidation (M)	77.063	248.25	Serotransferrin
P02790	3	3	6.3	Carbamidomethylation	51.676	18.785	Hemopexin
P04040	2	2	5.3	Carbamidomethylation	59.755	13.433	Catalase
P04114	1	1	0.2	Carbamidomethylation	515.6	6.5645	Apolipoprotein B-100
P04406	2	2	9.6	Carbamidomethylation	36.053	11.604	Glyceraldehyde-3-phosphate dehydrogenase
P05109	1	1	11.8	Carbamidomethylation	10.834	8.5403	Protein S100-A8
P05155	2	2	3.6	Carbamidomethylation	55.154	11.908	Plasma protease C1 inhibitor
P0C0L5	3	3	2.2	Carbamidomethylation	192.75	18.845	Complement C4-B
P0DOY3	3	3	31.1	Carbamidomethylation	11.265	19.978	Ig lambda-1 chain C regions
P0DP03	2	2	18.8	Carbamidomethylation	12.947	13.858	Ig heavy chain V-III region CAM
P14923	4	4	7.8	Carbamidomethylation	81.744	25.276	Junction plakoglobin
P19652	2	1	8.5	Carbamidomethylation	23.602	6.5307	Alpha-1-acid glycoprotein 2
P19823	1	1	1.2	Carbamidomethylation	106.46	6.4399	Inter-alpha-trypsin inhibitor heavy chain H2
P25311	1	1	3.4	Carbamidomethylation	34.258	6.61	Zinc-alpha-2-glycoprotein
P32119	4	4	18.2	Carbamidomethylation	21.892	24.321	Peroxiredoxin-2
P63261	8	2	20.5	Carbamidomethylation	41.792	57.245	Actin, cytoplasmic 2
P68871	7	3	46.9	Carbamidomethylation	15.998	98.447	Hemoglobin subunit beta
P69905	6	6	42.3	Carbamidomethylation, Oxidation (M)	15.257	60.639	Hemoglobin subunit alpha
P81605	3	3	22.7	Carbamidomethylation	11.284	18.546	Dermcidin
Q5D862	1	1	0.5	Carbamidomethylation	248.07	7.6741	Filaggrin-2
Q5T749	6	6	10.4	Carbamidomethylation	64.135	41.99	Keratinocyte proline-rich protein
Q5VTE0	3	3	7.1	Carbamidomethylation	50.184	19.062	Putative elongation factor 1-alpha-like 3
Q86YZ3	6	6	7.8	Carbamidomethylation	282.39	39.137	Hornerin
Q8NE71	2	2	2.1	Carbamidomethylation	95.925	11.39	ATP-binding cassette sub-family F member 1

Supplementary Table 8B

Lung Cancer							
Protein ID	#Peptides	#Unique peptides	Sequence coverage [%]	PTM	Mol. weight (kDa)	Score	Description
P00738	10	10	27.3	Carbamidomethylation, Oxidation (M)	45.205	126.08	Haptoglobin
P00915	6	6	32.6	Carbamidomethylation	28.87	37.302	Carbonic anhydrase 1
P01009	5	5	12.9	Carbamidomethylation	46.736	31.917	Alpha-1-antitrypsin
P01023	18	18	14	Carbamidomethylation	163.29	125.89	Alpha-2-macroglobulin
P01024	21	21	15.4	Carbamidomethylation	187.15	178.13	Complement C3
P01834	2	2	34.6	Carbamidomethylation	11.765	18.095	Ig kappa chain C region
P01857	8	3	28.8	Carbamidomethylation	36.105	25.922	Ig gamma-1 chain C region
P01860	9	2	24.1	Carbamidomethylation	41.287	61.822	Ig gamma-3 chain C region
P01871	7	7	18.3	Carbamidomethylation	49.439	58.845	Ig mu chain C region
P01876	3	3	8.8	Carbamidomethylation	37.654	20.735	Ig alpha-1 chain C region
P02647	7	7	29.2	Carbamidomethylation, Oxidation (M)	30.777	52.296	Apolipoprotein A-I
P02671	11	11	15.2	Carbamidomethylation	94.972	72.975	Fibrinogen alpha chain
P02675	10	10	27.9	Carbamidomethylation	55.928	82.572	Fibrinogen beta chain
P02679	6	6	14.3	Carbamidomethylation	51.511	40.592	Fibrinogen gamma chain
P02763	3	2	15.9	Carbamidomethylation	23.511	18.42	Alpha-1-acid glycoprotein 1
P02766	1	1	8.8	Carbamidomethylation	15.887	6.5995	Transthyretin
P02768	40	35	53	Carbamidomethylation, Oxidation (M)	69.366	323.31	Serum albumin
P02787	24	24	35.7	Carbamidomethylation, Oxidation (M)	77.063	248.25	Serotransferrin
P02790	3	3	6.3	Carbamidomethylation	51.676	18.785	Hemopexin
P04406	2	2	9.6	Carbamidomethylation	36.053	11.604	Glyceraldehyde-3-phosphate dehydrogenase
P05109	1	1	11.8	Carbamidomethylation	10.834	8.5403	Protein S100-A8
P07355	3	3	8.8	Carbamidomethylation	38.604	18.474	Annexin A2
P0DOY3	3	3	31.1	Carbamidomethylation	11.265	19.978	Ig lambda-1 chain C regions
P0DP03	2	2	18.8	Carbamidomethylation	12.947	13.858	Ig heavy chain V-III region CAM
P14923	4	4	7.8	Carbamidomethylation	81.744	25.276	Junction plakoglobin
P15924	7	7	2.4	Carbamidomethylation	331.77	46.43	Desmoplakin
P19652	2	1	8.5	Carbamidomethylation	23.602	6.5307	Alpha-1-acid glycoprotein 2
P25311	1	1	3.4	Carbamidomethylation	34.258	6.61	Zinc-alpha-2-glycoprotein
P32119	4	4	18.2	Carbamidomethylation	21.892	24.321	Peroxiredoxin-2
P35326	2	2	43.1	Carbamidomethylation	7.9653	15.62	Small proline-rich protein 2A
P63261	8	2	20.5	Carbamidomethylation	41.792	57.245	Actin, cytoplasmic 2
P68871	7	3	46.9	Carbamidomethylation	15.998	98.447	Hemoglobin subunit beta
P69905	6	6	42.3	Carbamidomethylation, Oxidation (M)	15.257	60.639	Hemoglobin subunit alpha
P81605	3	3	22.7	Carbamidomethylation	11.284	18.546	Dermcidin
Q02413	4	4	4.8	Carbamidomethylation	113.75	23.733	Desmoglein-1
Q08554	3	3	3.9	Carbamidomethylation	99.986	17.977	Desmocollin-1
Q5D862	1	1	0.5	Carbamidomethylation	248.07	7.6741	Filaggrin-2
Q5T749	6	6	10.4	Carbamidomethylation	64.135	41.99	Keratinocyte proline-rich protein
Q5VTE0	3	3	7.1	Carbamidomethylation	50.184	19.062	Putative elongation factor 1-alpha-like 3
Q86YZ3	6	6	7.8	Carbamidomethylation	282.39	39.137	Hornerin
Q8NE71	2	2	2.1	Carbamidomethylation	95.925	11.39	ATP-binding cassette sub-family F member 1
Q96RW7	2	2	0.4	Carbamidomethylation	613.38	12.113	Hemicentin-1

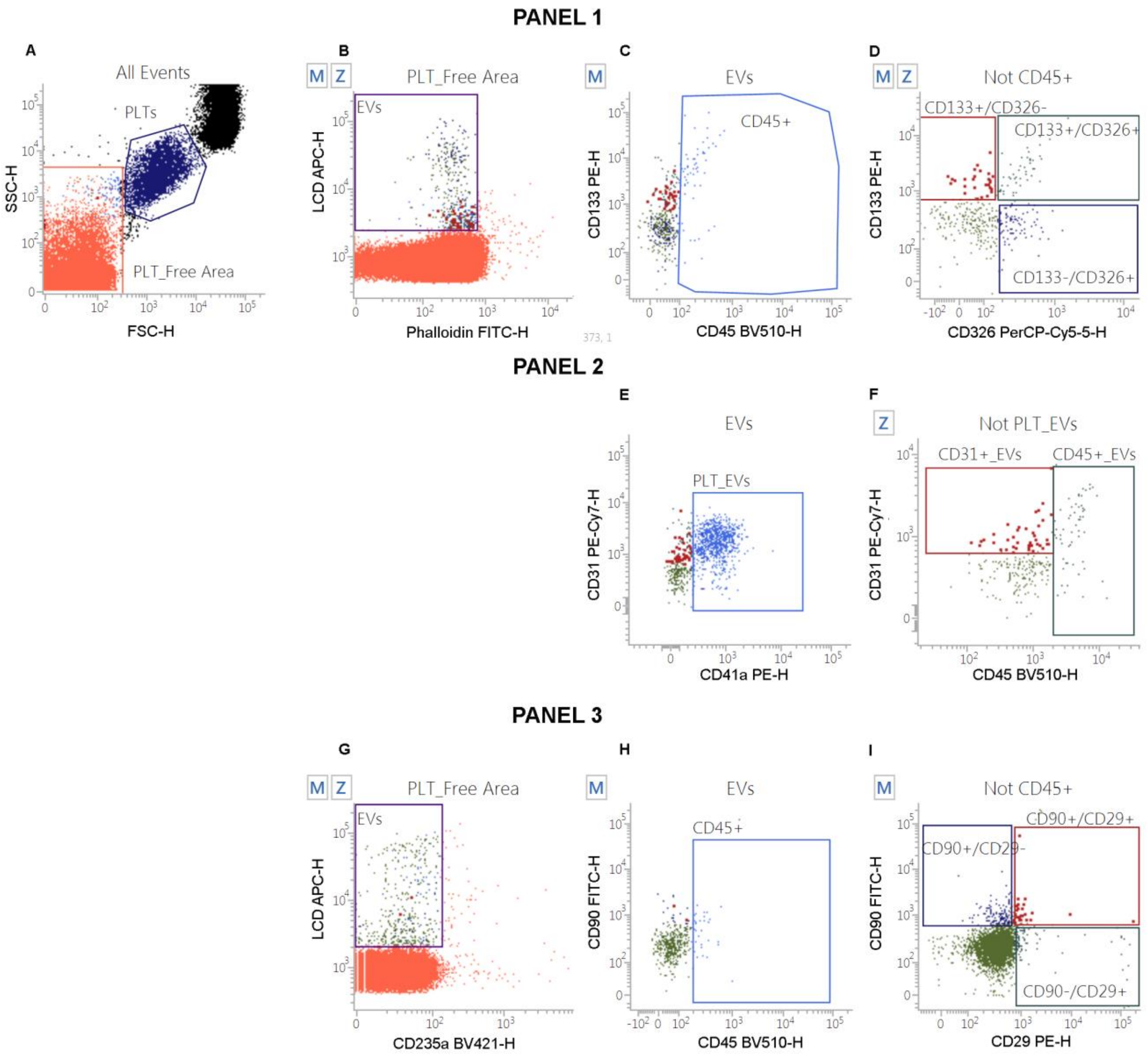
PANEL 1
A
All Events
PLTs
PLT_Free Area
SSC-H
FSC-H
B
M Z
PLT_Free Area
EVs
LCD APC-H
Phalloidin FITC-H
C
M
EVs
CD45+
CD133 PE-H
CD45 BV510-H
D
M Z
Not CD45+
CD133+/CD326-
CD133+/CD326+
CD133-/CD326+
CD133 PE-H
CD326 PerCP-Cy5-5-H
PANEL 2
E
EVs
PLT_EVs
CD31 PE-Cy7-H
CD41a PE-H
F
Z
Not PLT_EVs
CD31+_EVs
CD45+_EVs
CD31 PE-Cy7-H
CD45 BV510-H
PANEL 3
G
M Z
PLT_Free Area
EVs
LCD APC-H
CD235a BV421-H
H
M
EVs
CD45+
CD90 FITC-H
CD45 BV510-H
I
M
Not CD45+
CD90+/CD29+
CD90+/CD29-
CD90-/CD29+
CD90 FITC-H
CD29 PE-H

Supplementary Figure 1

Panel 1. **A**. A region ("platelet free area") under the one in which platelets (PLTs) fall was drawn on a SSC-H/FSC-H dot-plot. **B.** The "platelet free area" events were then represented on an LCD-H/Phalloidin-H dot-plot and EVs were identified as LCD positive/phalloidin negative dots. **C.** EVs (LCD+/Phalloidin- events) were analysed on a CD45-H/CD133-H dot-plot and CD45+ events were gated; a CD45 negative logical gate was then set and the resulting population was plotted on a (**D**) CD326-H/CD133-H dot-plot, where CD133+/CD326-, CD133+/CD326+, CD133-/CD326+ EVs were identified. **Panel 2**. **E.** EVs identified as shown in **A-B** were represented on a CD31-H/CD41a-H dot-plot, and events showing the CD31+/CD41a+ phenotype were identified as platelet-derived EVs (PLT-EVs); a PLT-EVs negative logical gate was set. **F.** The PLT-EV negative population was plotted on a CD45-H/CD31-H dot-plot, and CD45+ events were identified as leukocyte-derived EVs, while the CD31+/CD45- compartment was defined as the endothelial-derived EV population. **Panel 3**. **G.** The "platelet free area" events were identified as described in A, and then represented on an LCD-H/CD235a-H dot-plot; EVs were identified as LCD positive/CD235a negative dots. **H.** Those events were analysed on a CD45-H/CD90-H dot-plot and CD45+ events were gated. A CD45 negative logical gate was set. **I.** The resulting population was plotted on a CD29-H/CD90-H dot-plot, where CD90+/CD29-, CD90+/CD29+, and CD90-/CD29+ EVs were identified.

a)

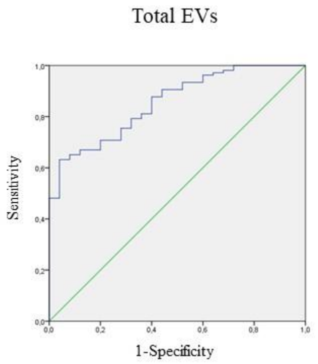

b)

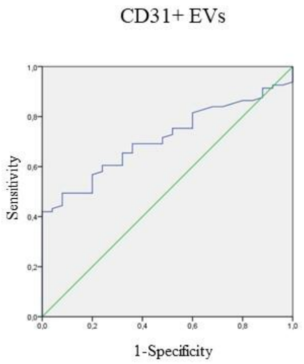

c)

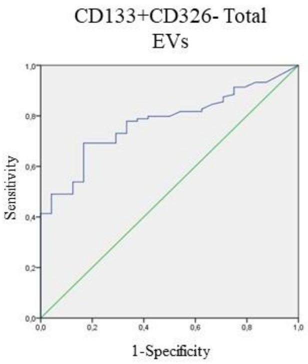

	Optimal cut-off (EV/µl)	Sensitivity	Specificity	AUC	Asintotic sig.
Total EV	10657.5	0.63	0.96	0.774	0.00003

	Optimal cut-off (EV/µl)	Sensitivity	Specificity	AUC	Asintotic sig.
CD31+EV	215.0	0.49	0.92	0.711	0.001

	Optimal cut-off (EV/µl)	Sensitivity	Specificity	AUC	Asintotic sig.
CD133+/ CD326- EV	82.5	0.69	0.84	0.774	0.00003

Supplementary Figure 2. ROC curves were calculated to determine the power of Total EV (a), CD31+ EV (b) and CD133+CD326- EV (c) concentrations as a discriminator of patients and healthy volunteers.

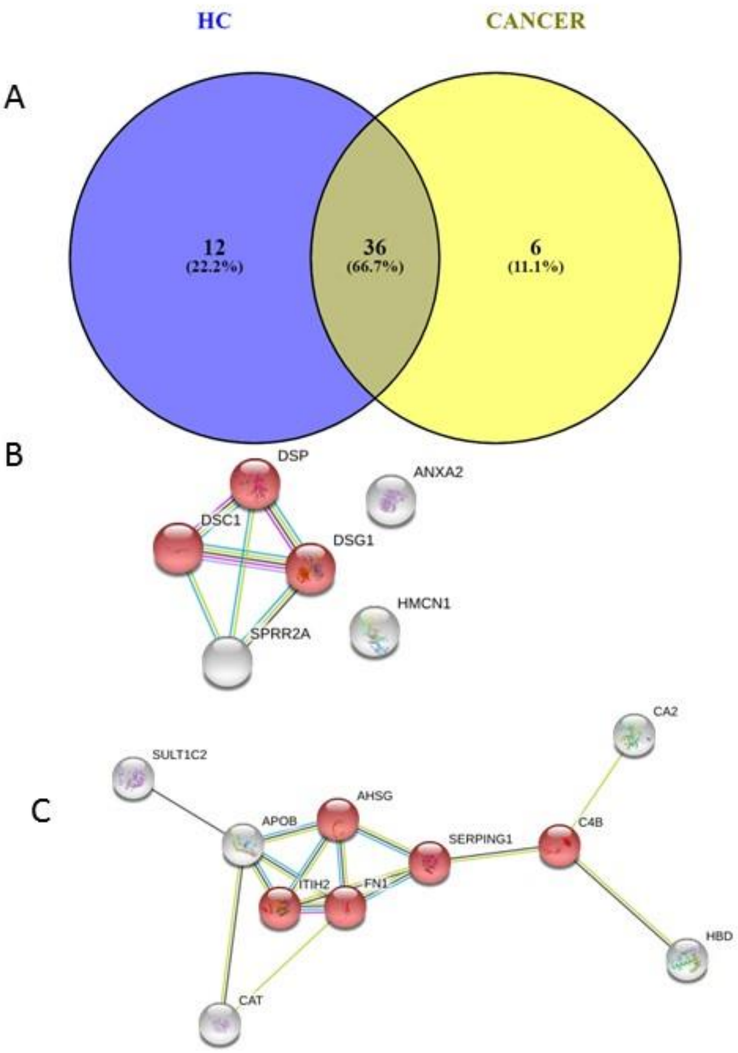

Supplementary Figure 3. A) Venn diagram of the identified proteins in Healthy Control (HC) pooled EVs and in lung cancer polled EVs. B) Three of the six proteins (reported as red dots) identified only in cancer EVs resulted associated to the "cell-cell adhesion" process (p = 0.0023). C) Ten proteins identified only in EVs from healthy volunteers resulted related to the "regulation of peptidase activity" (GO:0052547).
